# Supplementary material for: A comparative study of choroidal thickness and pigment epithelial detachment in acute and chronic central serous chorioretinopathy in Nepalese patients
Source: PLoS One. 2025 Nov 12;20(11):e0335947. doi: 10.1371/journal.pone.0335947 (PMC12611121; doi:10.1371/journal.pone.0335947)
Supplement: S3 Document — Metadata definitions and descriptions for CSCR eyes dataset variables. (DOCX) [file pone.0335947.s003.docx]

**Metadata**

**1. General Information**

- **Title**: Comparative Analysis of Choroidal Thickness and Pigment Epithelial Detachment in Acute and Chronic Central Serous Chorioretinopathy
- **Authors**:
  - Subash Bhatta¹
  - Nayana Pant¹
  - Suresh Raj Pant^2^
     (*¹Retina Department, Drishti Eye Hospital, ^2^Geta Eye Hospital)*
- **Date of Creation**: Date of dataset finalized: June 2023 Date of study finalized: Dec 2024
- **Description**:
  This dataset supports a study exploring the correlation of choroidal thickness and pigment epithelial detachment with disease activity in Central Serous Chorioretinopathy (CSCR) eyes. The study also provides dataset for average SFCT values in Nepalese population. It includes clinical records, Optical Coherence Tomography (OCT) imaging data, and results comparing acute and chronic CSCR cases to healthy controls.

**2. Content Information**

- **Keywords**: Central Serous Chorioretinopathy, Choroidal Thickness, Pigment Epithelial Detachment, OCT, Acute CSC, Chronic CSC
- **Variables/Attributes**:
  - **Sub foveal Choroidal Thickness (SFCT)**: Measured in micrometres across affected, fellow, and control eyes.
  - **Pigment Epithelial Detachment (PED)**: Observations of presence, type (flat/irregular or dome shaped/elevated), and distribution in acute and chronic cases.
  - **Central subfield thickness (CST)**: Statistical analysis of thickness values (measured in micrometres by OCT software).
  - **Patient Demographics**: Age, gender, diagnosis (acute/chronic), Chronic defined as Persistent and recurrent cases.
- **File Format:**  CSV (.csv)
- **Dataset Size**: [Dataset 1: Cases, 6 KB, and Dataset 2: Control, 3 KB].

**3. Data Collection Information**

- **Methods**:
  Data were collected via retrospective analysis of clinical records and OCT images from patients treated at a tertiary eye hospital in Nepal. Statistical tools included Chi-square test, Paired T-test, and Independent Sample T-test.
- **Time Period**: October 2021 to November 2022.
- **Geographical Location**: Nepal.

**4. Usage and Access**

- **License**: Creative Commons Attribution (CC BY)
- **Access Restrictions**: None
- **DOI or Unique Identifier**: [To be generated by the hosting platform, such as Zenodo or Figshare].

**5. Administrative Information**

- **Version**: Version 1.0 (or update as needed).
- **Contact Information**: subash.bhatta@gmail.com

**6. Supplementary Documentation**

- **Codebook**: Details on all variables and their coding (e.g., measurement units, statistical categorizations).

2 datasets: 1: Cases group: CSCR eyes, 2: Control group

1.First dataset of CSCR eyes

1. idno: Identfication number assigned to individual CSCR eyes. Total 145 CSCR eyes, 132 subjects, 119 subjects with single affected eyes, 26 both eyes affected cases are assigned ID no 1a to 13b, a and b being right and left eye
2. Study group (Categorical): 1. Case, 2. Control
3. Age (Continous): Age of CSCR cases
4. AGERANGE10 (Categorical): 1. 1-10, 2. 11-20, 3. 21-30, 4. 31-40, 5. 41-50, 6. 51-60, 7. 61-70
5. Sex (Categorical): Gender of CSCR cases. 1. Male, 2. Female
6. CT affected eye (Continous): Subfoveal choroidal thickness in CSCR eyes in μm
7. CT fellow eye(Continous): Subfoveal choroidal thickness in the fellow eye in CSCR cases who have CSCR in single eye in μm
8. CST(Continous): Central subfield thickness in μm
9. Eyes affected (Categorical): 1:Both eyes affected, 2: single eye affected
10. PEDYNAFF (Categorical): Pigment epithelial detachment in CSCR eyes: 1. Yes, 2. No
11. PED(Categorical): PED in CSCR eyes: 1. Dome shaped PED, 2. Absent, 3. Flat PED, 4. Mixed PED
12. PED other eye (Categorical): PED in the fellow eye of CSCR cases where single eye had CSCR 1. Dome shaped PED, 2. Absent, 3. Flat PED, 4. Mixed PED
13. Diagcat2 (Categorical): 1. Acute, 2. Persistent
14. Diagnosis (Categorical ): 1. Acute, 2. Chronic, 3. Recurrent

2.Second dataset of control group

1. Study group (Category): 1. Case, 2. Control
2. CT affected eye (Continous): Subfoveal choroidal thickness in right eye of control subjects in μm
3. CT fellow eye (Continous): Subfoveal choroidal thickness in left eye of control subjects in μm
4. Age (Continous): Age of control cases
5. AGERANGE10 (Categorical): 1. 1-10, 2. 11-20, 3. 21-30, 4. 31-40, 5. 41-50, 6. 51-60, 7. 61-70
6. Sex: (Categorical):Gender of control . 1. Male, 2. Female

- **Readme File**: Guidelines on dataset access, structure, and interpretation.

This dataset comprises two groups:

1. **CSCR Cases Group** – 145 CSCR eyes from 132 subjects diagnosed with Central Serous Chorioretinopathy (CSCR)
2. **Control Group** – Healthy age and gender matched 145 control subjects

**Dataset Access:**

The dataset is stored in two separate files: one for CSCR cases and one for controls. Names of files are

1. datasetCSCReyes.csv 2. datasetcontroleyes.csv

**Dataset Structure:**

**CSCR Dataset** includes variables such as age, sex, eye involved, choroidal thickness, macular thickness, PED, and diagnostic classifications.

**Control Dataset** includes similar structure focusing on age, sex, and choroidal thickness measurements in both eyes.

**Interpretation:**

- **Categorical variables** are numerically coded and explained in the codebook.
- **Continuous variables** include age in number and thickness measurements in micrometres (μm).

Please refer to the **Codebook** for interpretation of each variable and statistical categorization of variables.

- **Related Publications based on this dataset**: None
